# Alcohol and Cannabinoids – From the Editors

**DOI:** 10.35946/arcr.v42.1.10

**Published:** 2022-06-16

**Authors:** Jane Metrik, Sachin Patel

**Affiliations:** 1 Center for Alcohol and Addiction Studies, Brown University School of Public Health, Providence, Rhode Island; 2 Providence Veterans Affairs Medical Center, Providence, Rhode Island; 3 Department of Psychiatry and Behavioral Sciences, Northwestern University Feinberg School of Medicine, Chicago, Illinois

Alcohol is frequently used in association with cannabis, with co-use now perceived as normative with expanding cannabis legalization. Cannabinoid products are increasingly used for a number of medical and recreational purposes, including to enhance alcohol-reinforcing properties or in some cases to substitute for alcohol. Rates of alcohol use disorder (AUD) are higher among cannabis users relative to nonusers, with approximately 60% of individuals with current cannabis use disorder also meeting criteria for current AUD.^1^,^2^ Co-use is linked with heavy and problematic alcohol consumption, which in turn increases risk of alcohol-related diseases such as alcohol-associated liver disease. Co-use is also linked with a number of negative consequences, including behavioral risks,^3^ risk for driving safety, psychiatric comorbidity, adverse health effects, and poor alcohol treatment outcomes.^4^ However, the impact of cannabinoids on alcohol-related morbidity is not well understood, and findings on the impact of cannabis use on alcohol-related behaviors are equivocal. Cannabis serves both to complement drinking (i.e., increasing alcohol use), leading to more harmful consequences, and to substitute for alcohol effects (i.e., decreasing alcohol use and minimizing related risks).^5^ Beyond simultaneous (i.e., same-session) or temporally independent (e.g., same-week) co-use patterns, the substantial variability in cannabinoid composition (i.e., tetrahydrocannabinol [THC]-to-cannabidiol ratio), formulations (e.g., smoked, edibles), and quantity of cannabis could influence the direction of effect on alcohol-related outcomes. Further, individual differences associated with age and neurodevelopment; substance use disorders;^6^ motives for cannabis, alcohol, and simultaneous use;^7^ and the impact of state-level cannabis and alcohol regulatory policies^8^ could contribute to mixed findings on the risks and benefits of cannabinoids in relation to alcohol-related behaviors.

This research review series approaches cannabinoid–alcohol co-use through the lens of complex interactions between biological, psychological, and environmental factors. Basic science research reviewed in this topic series highlights the role of the endogenous cannabinoid or endocannabinoid (eCB) system in alcohol-related behaviors. The eCB system, which regulates cannabis reinforcement, is also involved in modulating alcohol reinforcement, motivation to consume alcohol, excessive alcohol consumption, AUD,^9^–^11^ and alcohol-related diseases. Emerging preclinical literature implicates exogenous cannabinoid receptor agonists (e.g., THC) in increased alcohol consumption, with chronic exposure to alcohol implicated in disruptions in eCB signaling.^12^,^13^ THC is the primary psychoactive constituent that interacts with the eCB system, producing intoxicating, rewarding, and reinforcing effects in a dose-dependent function. Although THC is the most commonly studied cannabinoid that defines cannabis potency, there are more than 100 other phytocannabinoids and more than 500 constituents in the cannabis plant that also may exert different effects on alcohol-related outcomes. For example, cannabidiol (CBD) is a nonpsychoactive, plant-based cannabinoid that has been implicated in the medicinal value of cannabis due to its potential antioxidant, anti-inflammatory, and analgesic effects.

Cannabinoids may reduce harmful effects of AUD, in part, by conferring beneficial effects on the gastrointestinal and immune systems.^14^–^16^ Endogenous cannabinoids, which are lipid molecules that exhibit cannabinoid-like properties, regulate various physiological functions in both the central nervous system and the peripheral organs, including the liver. Endocannabinoids and cannabinoid receptors in the liver modulate the progression of alcohol-related liver diseases via their effects on immune function, metabolic function, and inflammatory response.^17^ Preclinical research on the efficacy of eCB degradation inhibitors indicates that these inhibitors show promise as an emerging therapeutic target for AUD and cannabis use disorder treatment.^18^–^20^ Evidence from preclinical models also suggests CBD may have promise as a candidate pharmacotherapy for AUD.^21^ CBD attenuates proximal alcohol-related behaviors (e.g., preference, stress-induced alcohol seeking) and reduces alcohol consumption^22^,^23^ and alcohol-related physiological harms (e.g., liver steatosis and fibrosis, brain damage) in animal models.^24^–^26^ Overall, there is growing recognition of the therapeutic potential of the eCB system in reducing negative affective states associated with AUD and with abstinence from alcohol in AUD patients.^27^ Clinical studies on the acute and chronic impacts of specific cannabinoid and eCB targets on clinically relevant alcohol outcomes will help pave the way toward efficacious AUD pharmacotherapy and treatment of related medical conditions.

This translational research series strives to elucidate the cannabinoid–alcohol interactions by synthesizing findings across animal studies as well as human laboratory and epidemiological designs from community and clinical samples. From synapse to policy, the reviews in this series reflect the current state of the science on the reciprocal impact of alcohol and cannabinoids on an individual and the society at large. Several comprehensive reviews summarize findings from preclinical and human studies on the effects of alcohol exposure on the eCB system as a whole^28^ and more specifically at the synaptic level in the brain.^29^ In their review of the mechanisms of cannabinoid receptor signaling in hepatic pathogenesis, Yang, Choi, and Jeong summarize evidence in support of cannabinoid-based treatments for alcohol-associated liver disease.^30^ Lees, Debenham, and Squeglia present a comprehensive overview of longitudinal neuropsychological and neuroimaging studies on the independent and combined effects of cannabis and alcohol use on the developing human brain.^31^ Several articles review findings on the impact of cannabis use on alcohol consumption and consequences, and how this association may differ by cannabis formulation or by user characteristics,^32^ with a specific focus on simultaneous alcohol and cannabis use, and contextual characteristics of co-use in young adults.^33^ Finally, Pacula et al. provide a systematic review of published studies on the effect of liberalization of cannabis policies on alcohol use and co-use with cannabis in the United States and Canada.^34^

This topic series aligns with the research efforts discerning the shared impact of cannabinoids and alcohol on health undertaken by the Collaborative Research on Addiction at the National Institutes of Health (CRAN) partnership between the National Institute on Alcohol Abuse and Alcoholism, the National Institute on Drug Abuse (NIDA), and the National Cancer Institute. Elucidating effects of cannabis and alcohol co-use on health, policy, and economy is also a key research priority identified by the Cannabis Policy Research Workgroup of the NIDA National Advisory Council on Drug Abuse (NOT-DA-22-003). The empirical literature on cannabis and alcohol co-use has grown fourfold in the last decade alone, reflecting burgeoning interest in this topic. As summarized in the articles in this series, more research is needed to improve our understanding of the mechanisms underlying the functioning of eCBs in relation to alcohol in order to advance the development of eCB-based pharmacological treatments of AUD and related conditions. Clinical data examining the role of specific cannabinoids in alcohol-related human behavior also are critically needed to inform clinical guidelines for individuals engaged in AUD treatment and/or people who drink heavily and co-use cannabis. The authors lend crucial insights and make specific recommendations for future research endeavors on alcohol and cannabis interactions, taking into account between-person and within-person variability across time and contexts. All together, these findings will have important implications for the development of policy concerning alcohol in the context of the changing cannabis sociopolitical landscape.
